# The Burden of Chagas Disease in the Contemporary World: The RAISE Study

**DOI:** 10.5334/gh.1280

**Published:** 2024-01-08

**Authors:** Antonio Luiz Pinho Ribeiro, The RAISE Study Collaborators, Ísis Machado, Ewerton Cousin, Pablo Perel, Caroline Demacq, Yvonne Geissbühler, Aline de Souza, Alvaro Sosa Liprandi, Bruno R. Nascimento, Elisabeth F. França, Francisco Rogerlândio Martins-Melo, Gregory A. Roth, Israel Molina, Kenya Noronha, Lenice Ishitani, Mariângela Carneiro, Monica Quijano, Monica V. Andrade, Mohsen Naghavi, Jonathan F. Mosser, Daniel J. Piñeiro

**Affiliations:** 1 Hospital das Clínicas da Universidade Federal de Minas Gerais, Av. Prof. Alfredo Balena, 110 –Santa Efigênia, Belo Horizonte –MG, 30130-100, BR; 2 Department of Family Medicine, Mental and Collective Health, Universidade Federal de Ouro Preto, Ouro Preto, Brazil; 3 Institute for Health Metrics and Evaluation (IHME), University of Washington, Seattle, United States of America; 4 World Heart Federation, Geneva, Switzerland; 5 Department of Non-communicable Disease Epidemiology, London School of Hygiene & Tropical Medicine, London, United Kingdom; 6 Global Health, Novartis Pharma AG, Basel, Switzerland; 7 Evidence Generation, Innovative Medicines, Novartis Pharma AG, Basel, Switzerland; 8 Department of Economics, Faculty of Economic Sciences, Universidade Federal de Minas Gerais, Belo Horizonte, Brazil; 9 Cardiology Department, Sanatoria Guemes, Buenos Aires, Argentina; 10 Postgraduate Program in Public Health, Universidade Federal de Minas Gerais, Belo Horizonte, Brazil; 11 Instituto Federal de Educação, Ciência e Tecnologia do Ceará - Campus Fortaleza, Fortaleza, Brazil; 12 Department of Medicine, University of Washington, Seattle, United States of America; 13 International Health Unit Vall d’Hebron-Drassanes, Infectious Diseases Department, Vall d’Hebron University Hospital, Barcelona, Spain; 14 Epidemiological Surveillance Division, Belo Horizonte Municipal Health Department Belo Horizonte, Brazil; 15 Department of Parasitology, Universidade Federal de Minas Gerais, Belo Horizonte, Brazil; 16 Cardiology Department, Universidad de Buenos Aires, Buenos Aires, Argentina

**Keywords:** Chagas disease, Chagas cardiomyopathy, Burden of disease, Epidemiology

## Abstract

Chagas disease (ChD), a Neglected Tropical Disease, has witnessed a transformative epidemiological landscape characterized by a trend of reduction in prevalence, shifting modes of transmission, urbanization, and globalization. Historically a vector-borne disease in rural areas of Latin America, effective control measures have reduced the incidence in many countries, leading to a demographic shift where most affected individuals are now adults. However, challenges persist in regions like the Gran Chaco, and emerging oral transmission in the Amazon basin adds complexity. Urbanization and migration from rural to urban areas and to non-endemic countries, especially in Europe and the US, have redefined the disease’s reach. These changing patterns contribute to uncertainties in estimating ChD prevalence, exacerbated by the lack of recent data, scarcity of surveys, and reliance on outdated models.

Besides, ChD’s lifelong natural history, marked by acute and chronic phases, introduces complexities in diagnosis, particularly in non-endemic regions where healthcare provider awareness is low. The temporal dissociation of infection and clinical manifestations, coupled with underreporting, has rendered ChD invisible in health statistics. Deaths attributed to ChD cardiomyopathy often go unrecognized, camouflaged under alternative causes.

Understanding these challenges, the RAISE project aims to reassess the burden of ChD and ChD cardiomyopathy. The project is a collaborative effort of the World Heart Federation, Novartis Global Health, the University of Washington’s Institute for Health Metrics and Evaluation, and a team of specialists coordinated by Brazil’s Federal University of Minas Gerais. Employing a multidimensional strategy, the project seeks to refine estimates of ChD-related deaths, conduct systematic reviews on seroprevalence and prevalence of clinical forms, enhance existing modeling frameworks, and calculate the global economic burden, considering healthcare expenditures and service access. The RAISE project aspires to bridge knowledge gaps, raise awareness, and inform evidence-based health policies and research initiatives, positioning ChD prominently on the global health agenda.

## The Changing Landscape of Chagas Disease Epidemiology

Chagas disease (ChD) is a Neglected Tropical Disease (NTD) that affects impoverished communities and causes devastating health, social, and economic consequences [1,2]. Historically, vector-borne transmission of *Trypanosoma cruzi* was the main route of contagion. Other modes of transmission include blood/blood products, mother-to-child transmission, organ transplantation, and oral (food-borne) transmission. In recent decades, effective measures for ChD control have been implemented in many Latin American countries, resulting in decreased vectorial transmission and reduced new acute cases and prevalence [3]. In endemic countries where the vectorial transmission has been controlled, most individuals with ChD are now adults or elderly. ChD often co-exists with other cardiac risk factors and chronic diseases such as diabetes, coronary artery disease, and hypertensive heart disease. ChD transmission has persisted in some regions, such as the Gran Chaco, due to the ineffectiveness of vector control strategies, or has emerged in others due to the presence of oral transmission, as in the Amazon basin.

ChD is endemic in all 21 continental Latin American countries, with the Pan American Health Organization (PAHO) estimating 6 million infected people in 2010 [4]. The most significant number of *T. cruzi*-infected individuals was in Argentina, Brazil, and Mexico, with Bolivia having the highest prevalence. The United States has around 300,000 people with *T. cruzi* infection, 68,000–122,000 in Europe, most cases occurring in immigrants from Latin American countries.

PAHO estimates are an update of a previous evaluation published in 2006 and are based mostly on theoretical modelling on newly notified human infections and the estimated number of people at risk of *T. cruzi* infection [4]. There are very few available recent population surveys and empirical data on the ChD prevalence. The impressive reduction in the number of infected people compared with previous estimates is reportedly associated with reducing the population at risk, according to the success of vector control. However, the disease is lifelong, and the presence of current infection reflects the population at risk by transmission time, which may have occurred decades before. The available estimates on the prevalence of ChD are outdated, frequently derived from modelling and with a high level of uncertainty.

Migratory movements from rural to urban areas have also changed the landscape of ChD epidemiology [5]. From 1950 to 2010, the proportion of people living in Latin American cities grew from 40% to more than 80%, and more than 30 million Latin American immigrants moved to the Global North, mainly in the United States and Europe. The imprecision of estimates in non-endemic countries is high since they are usually generated based on the putative number of Latin American immigrants living in these non-endemic countries and the prevalence of ChD in the country of origin.

## A Lifelong Natural History of an Invisible Disease

ChD typically has acute and chronic phases, with acute infection occurring during the first years of life and being generally mildly symptomatic [5]. The acute phase resolves spontaneously; people usually become asymptomatic and enter the chronic phase. Chagas cardiomyopathy (CCM) is the most severe clinical presentation of the chronic phase, causing heart failure, arrhythmias, heart blocks, and stroke [1]. Over one-third of infected individuals develop CCM over a lifetime. This figure likely comes from early studies about the ChD natural history conducted in hyperendemic rural populations with acute infection at a young age, but now most individuals with ChD are in their fourth decade of life or older. In endemic areas, the lifetime risk of CCM is much higher since the disease progresses continuously during the lifespan at a progression rate of 1–2% per year. Chronic sequelae of the disease take decades to manifest, frequently occurring when they have already moved from rural areas to cities or even non-endemic countries. The temporal dissociation of the infection and clinical manifestations make it challenging to recognise ChD cases, even in patients with typical cardiac and digestive symptoms.

The PAHO estimates that 70% of those affected are unaware they have been infected, and less than 10% of those *T. cruzi*-infected individuals receive timely diagnosis and effective treatment with antiparasitic drugs in the early stages of the disease [6]. Healthcare providers also have low awareness of ChD, especially in non-endemic countries, with a lack of knowledge on whom to screen and clarity on appropriate tests and clinical management [7,8]. For this reason, the theme of World Chagas Disease Day 2022 was ‘finding and reporting every case to defeat Chagas disease’.

Due to the limited awareness of the disease by both the population and health professionals, ChD is underreported as a cause of death for the death certificates, even for ChD patients who died due to heart disease [9]. Deaths related to CCM have been systematically reported in death certificates as other causes, including dilated cardiomyopathy, paroxysmal tachycardia, and heart failure [9]. This underreporting of ChD-related deaths further reduces its visibility in regional and national health statistics, turning this neglected disease unseen for health policymakers and other stakeholders. Beyond being neglected for affecting impoverished, voiceless people, ChD becomes neglected for being invisible to the population, healthcare providers, and health statistics.

## Raising the Profile of ChD

**Figure 1:**
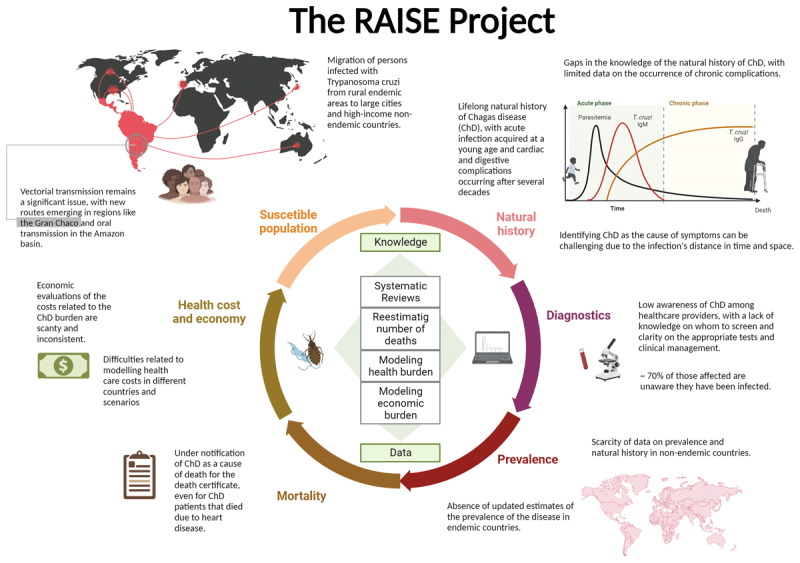
Main gaps in the knowledge and barriers to the estimation of the global health and economic burden of Chagas disease.

The Global Burden of Diseases, Injuries, and Risk Factors Study (GBD) provides standardised estimates for the burden of diseases by age, sex, location, and year. Challenges in modelling ChD include extreme geographical heterogeneity, rapidly evolving temporal trends, decades-long lag between infection and symptomatic disease, biased prevalence data, incomplete recognition of ChD-attributable deaths, limited data on sequela, and a near-total absence of data outside endemic countries (Figure 1) [10]. ChD and CCM carry a substantial social and economic burden, which is also largely unknown.

To better understand these issues, we launched the RAISE project, which stands for ‘The buRden of ChAgas dISEase in the contemporary world’. Based on current estimates of prevalence, mortality, and the frequency of complications, we will review current knowledge and calculate the burden of ChD and CCM. We also intend to estimate the costs associated with these conditions and specific complications. The project is a collaborative effort of the World Heart Federation, Novartis Global Health, the University of Washington’s Institute for Health Metrics and Evaluation, and a team of ChD specialists coordinated by Brazil’s Federal University of Minas Gerais. This multi-part descriptive study will include systematic reviews and disease modelling to obtain plausible estimates of the disease’s health and economic burden. Where applicable, this work will follow relevant reporting guidelines such as PRISMA and GATHER.

To accomplish this task, we will follow a multidimensional strategy. First, we will re-estimate the number of deaths due to ChD. Where data allow, we will perform empirical studies in vital registration datasets, using methods to evaluate the misclassification of death and redistribution of ill-defined and garbage codes, that is, International Classification of Diseases (ICD)-10 codes used in death certificates as the underlying cause of death which are not sufficiently precise to be useful for public health intervention or are simply implausible.

Second, we will conduct systematic reviews of the seroprevalence of ChD in endemic and non-endemic countries to improve the evidence base for estimating ChD incidence, prevalence, and morbidity. Also, we will conduct a systematic review of the clinical forms of ChD to estimate better the prevalence of different sequelae of ChD by age and sex.

Third, we will review and enhance the fatal and non-fatal GBD ChD modelling framework used in the Global Burden of Disease study, incorporating these updated data in the analysis. Then, we will estimate the global burden of ChDs for endemic and non-endemic countries measured by deaths, YLLs (years of life lost to due to premature mortality), prevalence, YLDs (years lived with a disability), and DALYs (disability-adjusted life years).

Fourth, we will estimate the global and country-specific economic burden of ChD and CCM, including those related to their main cardiac manifestations and non-drug-related treatments (e.g., transplants, pacemakers, cardiac devices), from the societal perspective. The modelling step will be preceded by a systematic review of the available literature on the costs of ChD treatment. We will use a Markovian model for groups of countries classified according to the total healthcare expenditures per capita and level of coverage of the public healthcare system. Compared with the previous analysis, our estimation will consider a more comprehensive set of countries and distinguish them by differences in access to healthcare services.

We hope that the results of this effort will help break the epidemiological silence related to ChD and to reposition this neglected disease in the list of priorities of governments and international organisations. Combining up-to-date data on prevalence, mortality, cause of death reclassification approaches, and economic modelling will allow more accurate knowledge of ChD burden and point towards innovative strategies for future planning of health policies and research initiatives. Raising the profile of ChD and bridging the existing knowledge gaps through a broad approach are important steps for eliminating this ominous and dreadful disease.

## The RAISE Study Collaborators

Antonio Luiz P Ribeiro, MD^a^, Ísis Machado, PhD^b^, Ewerton Cousin, PhD^c^, Pablo Perel, MD^d,e^, Caroline Demacq, PhD^f^, Yvonne Geissbühler, PhD^g^, Aline de Souza, MSc^h^, Alvaro Sosa Liprandi, MD^i^, Bruno R. Nascimento, MD^a^, Elisabeth França, MD^j^, Francisco Rogerlândio Martins-Melo, PhD^k^, Gregory A Roth, MD^c,l^, Israel Molina, PhD^m^, Kenya Noronha, PhD^h^, Lenice Ishitani, PhD^n^, Mariângela Carneiro^o^, Monica Quijano, MD^f^, Monica V Andrade, PhD^h^, Mohsen Naghavi, MD^c^, Jonathan F Mosser, MD^c^, Daniel J. Piñeiro, MD^d,p^

^a^ Department of Internal Medicine, Faculdade de Medicina, and Telehealth Center and Cardiology Service, Hospital das Clínicas, Universidade Federal de Minas Gerais, Belo Horizonte, Brazil

^b^ Department of Family Medicine, Mental and Collective Health, Universidade Federal de Ouro Preto, Ouro Preto, Brazil

^c^ Institute for Health Metrics and Evaluation (IHME), University of Washington, Seattle, United States of America

^d^ World Heart Federation, Geneva, Switzerland

^e^ Department of Non-communicable Disease Epidemiology, London School of Hygiene & Tropical Medicine, London, United Kingdom

^f^ Global Health, Novartis Pharma AG, Basel, Switzerland

^g^ Evidence Generation, Innovative Medicines, Novartis Pharma AG, Basel, Switzerland

^h^ Department of Economics, Faculty of Economic Sciences, Universidade Federal de Minas Gerais, Belo Horizonte, Brazil

^i^ Cardiology Department, Sanatoria Guemes, Buenos Aires, Argentina

^j^ Postgraduate Program in Public Health, Universidade Federal de Minas Gerais, Belo Horizonte, Brazil

^k^ Instituto Federal de Educação, Ciência e Tecnologia do Ceará - Campus Fortaleza, Fortaleza, Brazil

^l^ Department of Medicine, University of Washington, Seattle, United States of America

^m^ International Health Unit Vall d’Hebron-Drassanes, Infectious Diseases Department, Vall d’Hebron University Hospital, Barcelona, Spain

^n^ Epidemiological Surveillance Division, Belo Horizonte Municipal Health Department Belo Horizonte, Brazil

^o^ Department of Parasitology, Universidade Federal de Minas Gerais, Belo Horizonte, Brazil

^p^ Cardiology Department, Universidad de Buenos Aires, Buenos Aires, Argentina
